# Mechanical Changes and Microfilament Reorganization Involved in Microcystin-LR-Promoted Cell Invasion in DU145 and WPMY Cells

**DOI:** 10.3389/fphar.2020.00089

**Published:** 2020-02-26

**Authors:** Qiang Zhang, Guihua Wang, Yongfang Xie, Zhiqin Gao, Zumu Liang, Zhifang Pan, Guohui Wang, Weiguo Feng

**Affiliations:** ^1^ College of Bioscience and Technology, Weifang Medical University, Weifang, China; ^2^ Department of Fundamental Veterinary, College of Veterinary Medicine, Shandong Agricultural University, Tai’an, China

**Keywords:** microcystin-LR, mechanical parameters, invasion, microfilament, microfilament-associated protein

## Abstract

Microcystin-leucine arginine (MC-LR) is a potent tumor initiator that can induce malignant cell transformation. Cellular mechanical characteristics are pivotal parameters that are closely related to cell invasion. The aim of this study is to determine the effect of MC-LR on mechanical parameters, microfilament, and cell invasion in DU145 and WPMY cells. Firstly, 10 μM MC-LR was selected as the appropriate concentration *via* cell viability assay. Subsequently, after MC-LR treatment, the cellular deformability and viscoelastic parameters were tested using the micropipette aspiration technique. The results showed that MC-LR increased the cellular deformability, reduced the cellular viscoelastic parameter values, and caused the cells to become softer. Furthermore, microfilament and microfilament-associated proteins were examined by immunofluorescence and Western blot, respectively. Our results showed that MC-LR induced microfilament reorganization and increased the expression of p-VASP and p-ezrin. Finally, the impact of MC-LR on cell invasion was evaluated. The results revealed that MC-LR promoted cell invasion. Taken together, our results suggested that mechanical changes and microfilament reorganization were involved in MC-LR-promoted cell invasion in DU145 and WPMY cells. Our data provide novel information to explain the toxicological mechanism of MC-LR.

## Introduction

Microcystins (MCs) are a group of cyclic heptapeptide toxins produced by freshwater cyanobacteria (Rastogi et al., 2014), among which microcystin-leucine arginine (MC-LR) is the most abundant and toxic congener (Li Y. et al., 2016). The World Health Organization (WHO) has recommended 1 µg/L as the upper limit of MC-LR in drinking water (Wang et al., 2010). In fact, the concentration of MC-LR in the drinking water has exceeded this recommend value in many countries (Backer et al., 2010; Duong et al., 2014; Chun et al., 2018). Current studies show that MC-LR induces cytotoxicity in different ways. In detail, it has been shown that MC-LR can inhibit serine/threonine protein phosphatases (PPs) by interactions with their catalytic subunits, then affecting the cellular homeostasis (Zhou et al., 2015; Liu et al., 2016). Also, MC-LR can induce oxidative stress by increasing the reactive oxygen species (ROS) or decreasing the glutathione (GSH). Subsequently, oxidative stress caused by MC-LR might induce mitochondrial permeability transition and apoptosis (Ma et al., 2016; Ma et al., 2018; Wu et al., 2019). In addition, previous studies indicated that MC-LR is a potent tumor initiator (Zhao et al., 2016; Zhu et al., 2018), which could induce malignant cell transformation. For example, Liu et al. reported that MC-LR promoted proliferation and inhibited apoptosis in normal human liver cells (HL7702) (Liu et al., 2016). The study of Wang et al. showed that MC-LR induced cytoskeleton reorganization, resulting in increased cell migration in human laryngeal epithelial cells (Hep-2) (Wang et al., 2017). However, the effect of MC-LR on prostate cancer (PCa) cells and normal prostate cells has yet been studied.

Cellular mechanical characteristics are pivotal parameters that can reveal the properties of cells, such as their proliferation, migration, and invasion (Yin-Quan et al., 2014). It has been reported that the viscoelasticity of cancer cells decreased significantly and was closely related to their metastasis and invasion (Rother et al., 2014; Runge et al., 2014; Nguyen et al., 2016). To date, the effect of MC-LR on cellular mechanical properties is unknown. In addition, microfilaments are the main components of the cytoskeleton in the cytoplasm. Many studies have suggested that MC-LR can cause microfilament changes in different cells (Meng et al., 2011; Zeng et al., 2015; Wang et al., 2017). However, the influence of MC-LR on microfilaments is still poorly documented in PCa cells and normal prostate cells.

The aim of this study is to determine the effect of MC-LR on mechanical parameters, microfilament, and phenotype in PCa cells and normal prostate cells. To do this, we incubated PCa cells (DU145) and normal prostate cells (WPMY) with 10 μM MC-LR and then tested the cellular deformability and viscoelastic parameters by the micropipette aspiration technique. Subsequently, the changes of microfilament and microfilament-associated proteins were examined by immunofluorescence and Western blot, respectively. Moreover, invasion assay was conducted to assess the role of MC-LR in cell invasion.

## Materials and Methods

### Cell Culture and Treatment

DU145 and WPMY cells were obtained from the Chinese Academy of Sciences Cell Bank (Shanghai, China) and cultured in Dulbecco’s modified Eagle’s medium (DMEM; Invitrogen, Shanghai, China) supplemented with 10% fetal bovine serum (FBS; Gibco, Shanghai, China). After 24 h of incubation, cells were treated with MC-LR (Express Technology, Beijing, China) for another 24 h. The control cells were cultured in medium without MC-LR.

### CCK-8 Assay

DU145 and WPMY cells were seeded in 96-well plates, respectively. After treatment with different concentrations of MC-LR (0.1 μM, 0.5 μM, 1 μM, 2 μM, 5 μM, 10 μM, 20 μM, and 40 μM) for 24 h, relative cell viability was detected by the CCK-8 kit (Beyotime, Shanghai, China) according to the manufacturer’s instructions (Wang et al., 2017).

### Micropipette Aspiration Test and the Mechanical Behavior of Cells

**Figure 1:**
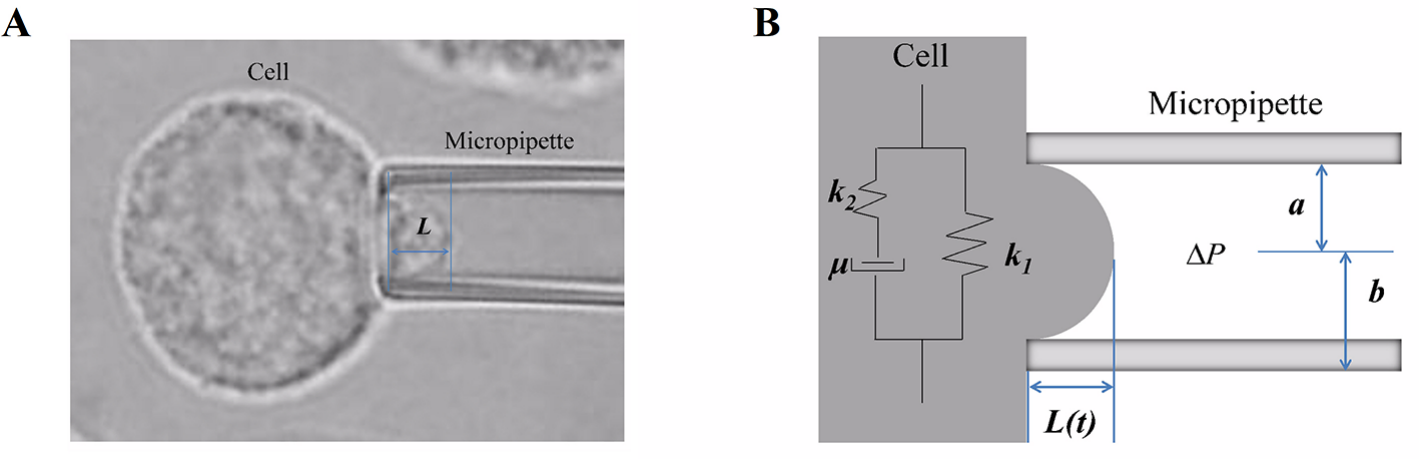
The micropipette aspiration technique and the mechanical behavior of cells. **(A)** A certain negative pressure (ranging from 294 Pa to 441 Pa) induced cell deformation with time, and then, part of the cell was aspirated into the micropipette. Images of this process were recorded, and the aspirated length *L* was measured. **(B)** Schematic representation of the Kelvin standard linear viscoelastic solid model: in the model, the cell was assumed to be a homogeneous viscoelastic spherical solid; *k_1_, k_2_*_,_ and *μ* are the viscoelastic parameters, *L* is the aspirated length, and *Δp* is the negative pressure.

The mechanical behaviors of cells were represented by the deformability and viscoelastic parameters, which were investigated using the micropipette aspiration technique combined with the Kelvin standard linear viscoelastic solid model (Xie et al., 2019) (**Figure 1**). A single-cell suspension was prepared for the micropipette aspiration test by micromanipulator (Olympus, Japan). A single spherical cell was captured and deformed by the negative pressure in the micropipette, and then part of the cell was aspirated into the micropipette. This process was viewed and recorded with an inversion microscope (Olympus, Japan) combined with image software. The recorded images of the micropipette aspiration test were used to measure the aspirated length of cells (**Figure 1A**), and then the relationships of time and aspirated length were obtained and fitted to calculate the cellular viscoelastic parameter values.

The cell was assumed to be a homogeneous viscoelastic spherical solid, and then the cellular viscoelastic parameters *E_0_* (the instantaneous modulus), *E_∞_* (the equilibrium modulus associated with long-term equilibrium), and *μ* (the apparent viscosity) were calculated by applying the Kelvin standard linear viscoelastic solid model (**Figure 1B**) based on the relationships of time-aspirated length, as our (Xie et al., 2019) and others’ (Zhang et al., 2008) previous studies have described. The values of the cellular viscoelastic parameters (*E_0_*, *E_∞_*, and *μ*) were calculated according to the following equation:

$$\begin{array}{c} L(t)=\frac{3a\text{Δ}p}{\pi E_{\infty }}\times \left[1+\left(\frac{k_{1}}{k_{1}+k_{2}}-1\right)\exp\left(-\frac{t}{\tau }\right)\right]\\ \mu =\frac{\tau \cdot k_{1}\cdot k_{2}}{k_{1}+k_{2}},E_{0}=\frac{3}{2}(k_{1}+k_{2}),E_{\infty }=\frac{3}{2}k_{1} \end{array}$$

### Immunofluorescence

After 24-h treatment with MC-LR, immunofluorescence was performed to detect microfilament changes in DU145 and WPMY cells as previously described (Huang et al., 2018). Briefly, the cells were fixed using 4% formaldehyde for 15 min and then washed by phosphate-buffered saline (PBS). Subsequently, phalloidin (Beyotime, Shanghai, China) and DAPI (Santa Cruz, Dallas, USA) were added and then incubated for 60 min. The images were captured using an Olympus laser-scanning confocal microscope.

### Western Blot

Based on a previous study (You et al., 2017), the process of Western blot was slightly modified. After SDS-PAGE, the protein was transferred to PVDF membranes (Beyotime, Shanghai, China) and incubated with anti-VASP (CST, Boston, USA, 1:1000 dilution), anti-ezrin (CST, Boston, USA, 1:1000 dilution), anti-p-VASP (Ser157, CST, Boston, USA, 1:1000 dilution), anti-p-ezrin (Thr567, CST, Boston, USA, 1:1000 dilution), and anti-β-actin (Beyotime, Shanghai, China, 1:1000 dilution) at 4°C overnight. After incubating with secondary antibody (Proteintech, Wuhan, China, 1:2000 dilution), the bands were analyzed using the enhanced chemiluminescence reaction kit (ECL; Beyotime, Shanghai, China).

### Invasion Assay

Invasion assay was carried out using the method previously described (Yan et al., 2018). DU145 and WPMY cells were seeded into transwell chambers (Sigma-Aldrich, St. Louis, USA), respectively. Meanwhile, serum-free DMEM with 10 μM MCLR was added to the upper chamber, and DMEM containing 10% FBS was added to the lower chamber. After incubating for 24 h, the cells that passed through the membranes were stained with 0.5% crystal violet solution and counted under the microscope.

### Statistical Analysis

Statistical analyses were performed with SPSS 19.0 software. Independent sample t-test was used to determine the differences between groups. All experiments were performed in triplicate. Data are shown as mean ± SD, and P < 0.05 was considered to be significantly different.

## Results

### Screening of MC-LR Concentration

**Figure 2:**
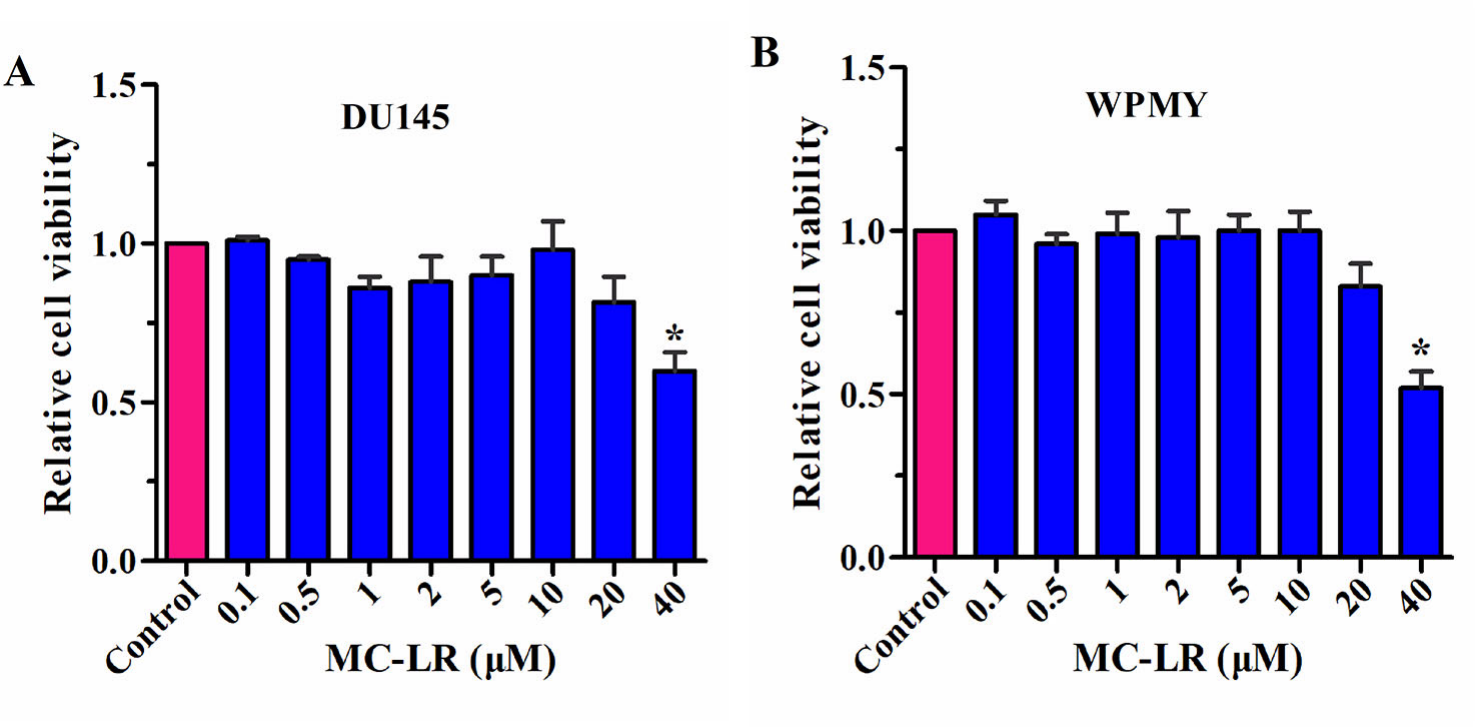
Screening of MC-LR concentration. DU145 cells and WPMY cells were seeded in 96-well plates, respectively. After incubation with different concentrations of MC-LR for 24 h, relative cell viability was detected with the CCK-8 kit. The results are representative of three independent experiments. *P < 0.05. Error bars indicate SD.

To select the appropriate concentration of MC-LR, a CCK-8 kit was used to detect the relative cell viability. As shown in **Figure 2**, no change was observed in the relative cell viability of DU145 and WPMY cells between the control group and the MC-LR treated group with concentrations of 0.1-10 μM. However, the relative cell viability decreased at an MC-LR concentration of 20 μM and more so at 40 μM. Therefore, 10 μM MC-LR was selected for further study.

### MC-LR Caused Mechanical Behavior Changes in DU145 and WPMY Cells

**Figure 3:**
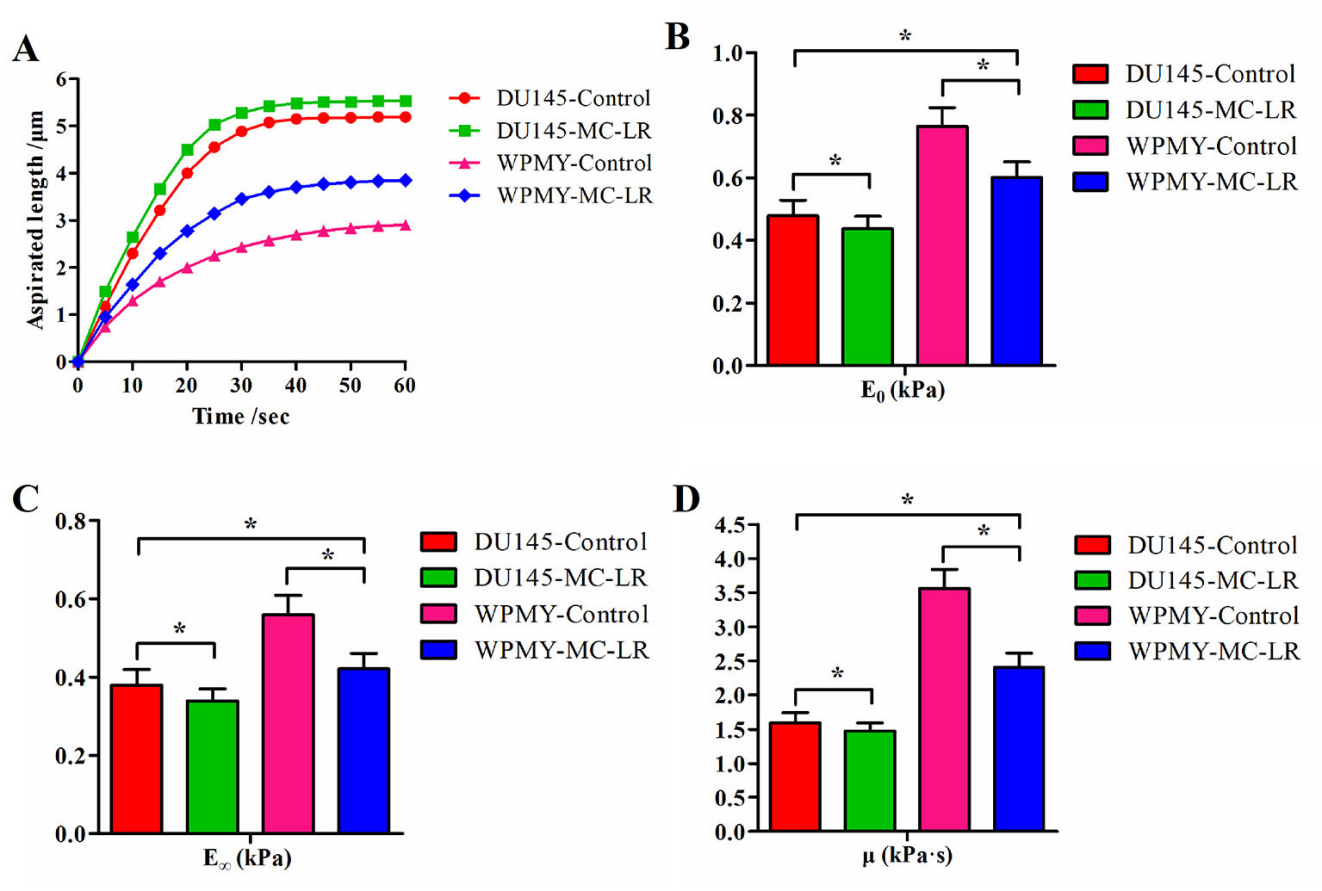
MC-LR caused mechanical behavior changes in DU145 cells and WPMY cells. **(A)** Curves of aspirated lengths *L* with time at a constant negative pressure of 392 Pa. **(B**–**D)** Comparison of the cellular viscoelastic parameters (*E_0_*, *E_∞_*, and *μ*). The viscoelastic parameter values were calculated by applying the curves of the aspirated lengths and the Kelvin standard linear viscoelastic solid model. The results are representative of three independent experiments. *P < 0.05. Error bars indicate SD.

The relationships between time and the aspirated length of the cells were plotted as curves, and the time–aspirated length curves at a negative pressure of 392 Pa are shown in **Figure 3A**. Under the negative pressure, the cell was deformed; meanwhile, part of the cell was aspirated into the micropipette, and the deformation rate decreased with time until it was no longer aspirated into the micropipette within 50-60 sec. The time–aspirated length curves of the cells reflected the cellular deformability. As shown in **Figure 3A**, MC-LR treated cells exhibited higher deformability than MC-LR untreated cells. These results suggested that MC-LR increased the deformability of the cells. In addition, DU145 cells showed higher deformability than WPMY cells, and DU145 cells without MC-LR treatment even still exhibited higher deformability than WPMY cells treated with MC-LR.

**Figures 3B–D** show comparisons of the cellular viscoelastic parameters (*E_0_*, *E_∞_*, and *μ*), respectively. The viscoelastic parameter values (*E_0_*, *E_∞_*, and *μ*) of MC-LR treated cells were significantly lower than those of the untreated cells (P < 0.05). Additionally, the viscoelastic parameter values (*E_0_*, *E_∞_*, and *μ*) of DU145 cells were lower than those of WPMY cells (P < 0.05). These results indicated that the prostate carcinoma cells were softer than normal prostate cells and that MC-LR induced the cells to become much softer. Previous research indicated that cellular mechanical behavior is correlated with microfilament changes. Hence, we investigated the microfilament arrangement further.

### MC-LR Induced Microfilament Reorganization in DU145 Cells and WPMY Cells

**Figure 4:**
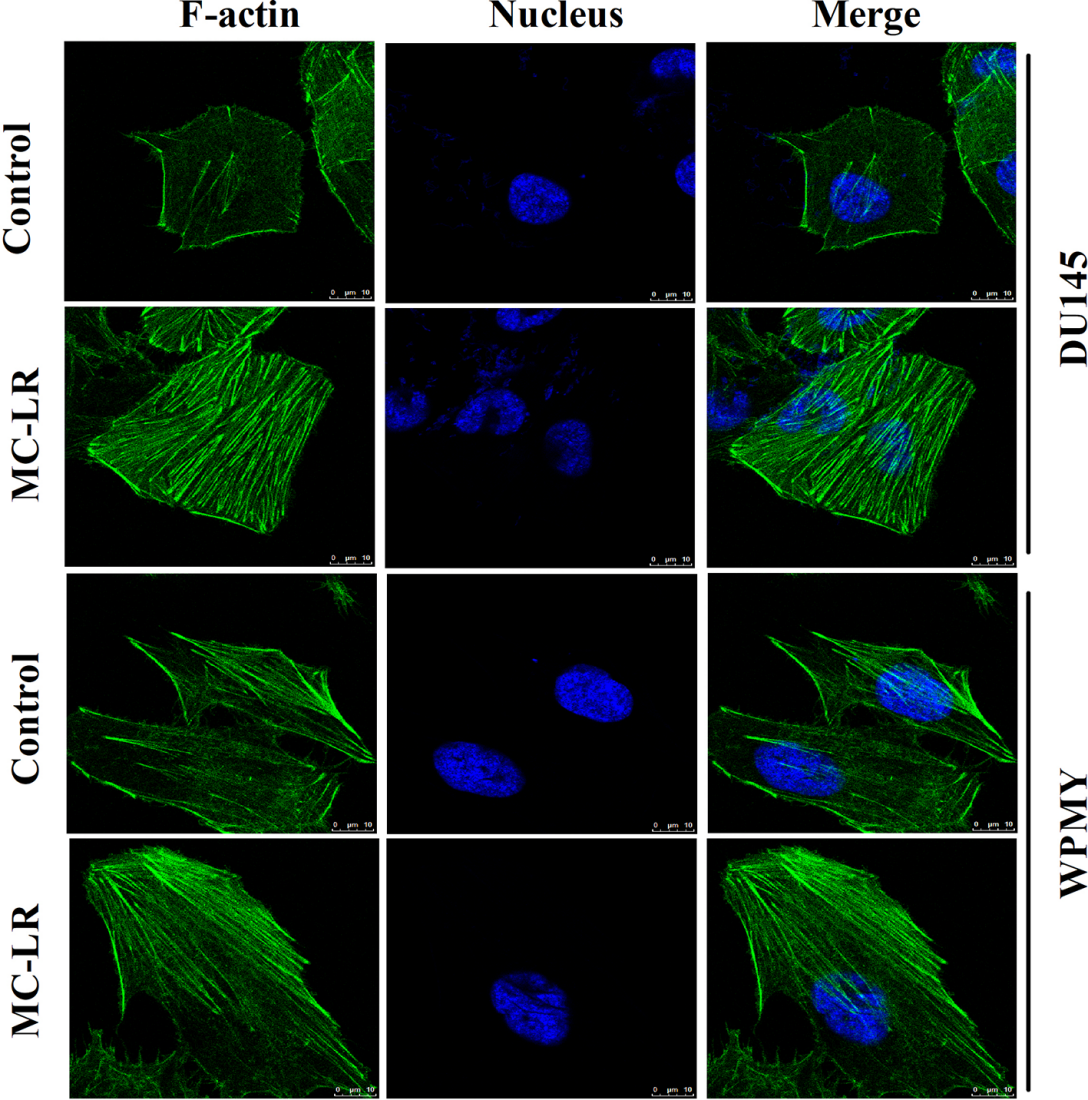
MC-LR induced microfilament reorganization in DU145 cells and WPMY cells. DU145 cells and WPMY cells were incubated using 10 μM MC-LR for 24 h, and then immunofluorescence was performed to label microfilament (green) and nuclei (blue), respectively. The results are representative of three independent experiments.

Changes of microfilament were detected in DU145 and WPMY cells after MC-LR treatment. As shown in **Figure 4**, microfilament was evenly distributed in the inner side of the cell membranes in the control group; by contrast, in the MC-LR treated group, microfilament appeared to gather to the cell surface and concentrated to form bundles. Notably, the above results were observed in both DU145 and WPMY cells. These data suggested that MC-LR induced microfilament reorganization in both DU145 and WPMY cells.

### MC-LR Increased the Phosphorylation Level of Microfilament-Associated Proteins in DU145 and WPMY Cells

**Figure 5:**
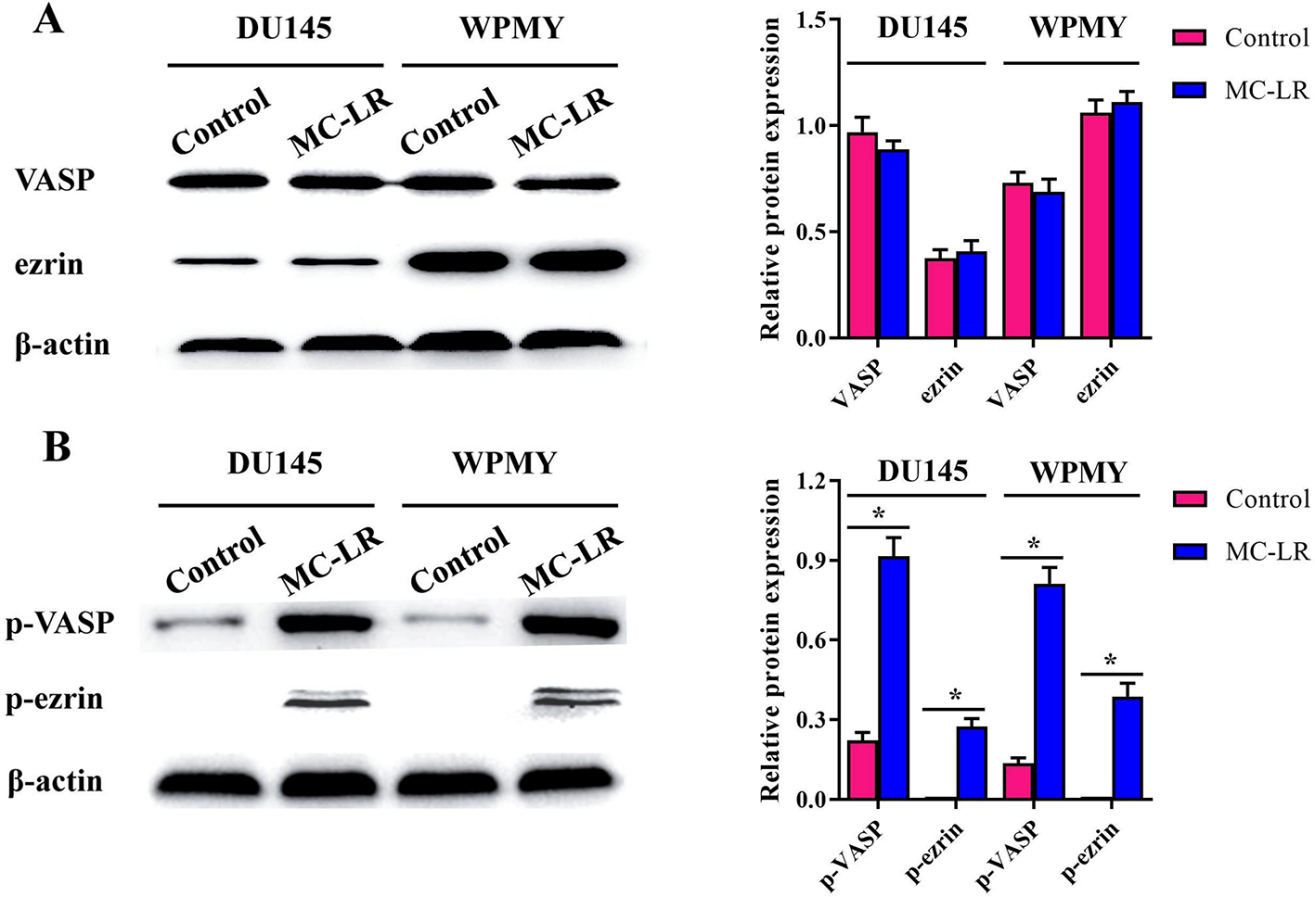
MC-LR increased the phosphorylation level of microfilament-associated proteins in DU145 cells and WPMY cells. DU145 cells and WPMY cells were incubated with 10 μM MC-LR for 24 h, and then Western blot was carried out to detect the expression and phosphorylation of VASP and ezrin. The results are representative of three independent experiments. * *P* < 0.05. Error bars indicate SD.

To further elucidate the mechanism of microfilament rearrangement, the expression and phosphorylation of microfilament-associated proteins were detected by Western blot. Firstly, the expression of microfilament-associated proteins (VASP and ezrin) was tested in DU145 and WPMY cells after MC-LR treatment. As shown in **Figure 5A**, no significant change was observed in the expression of VASP and ezrin, which implied that microfilament reorganization had no relationship with the expression of microfilament-associated proteins. Subsequently, the phosphorylation level of microfilament-associated proteins was further assessed. The results showed that the expression of p-VASP (Ser157) and p-ezrin (Thr567) was significantly higher in the MC-LR treatment group than in the untreated group (P < 0.05, **Figure 5B**). These results demonstrated that MC-LR increased the phosphorylation level of microfilament-associated proteins in DU145 and WPMY cells.

### MC-LR Promoted Cell Invasion in DU145 and WPMY Cells

**Figure 6:**
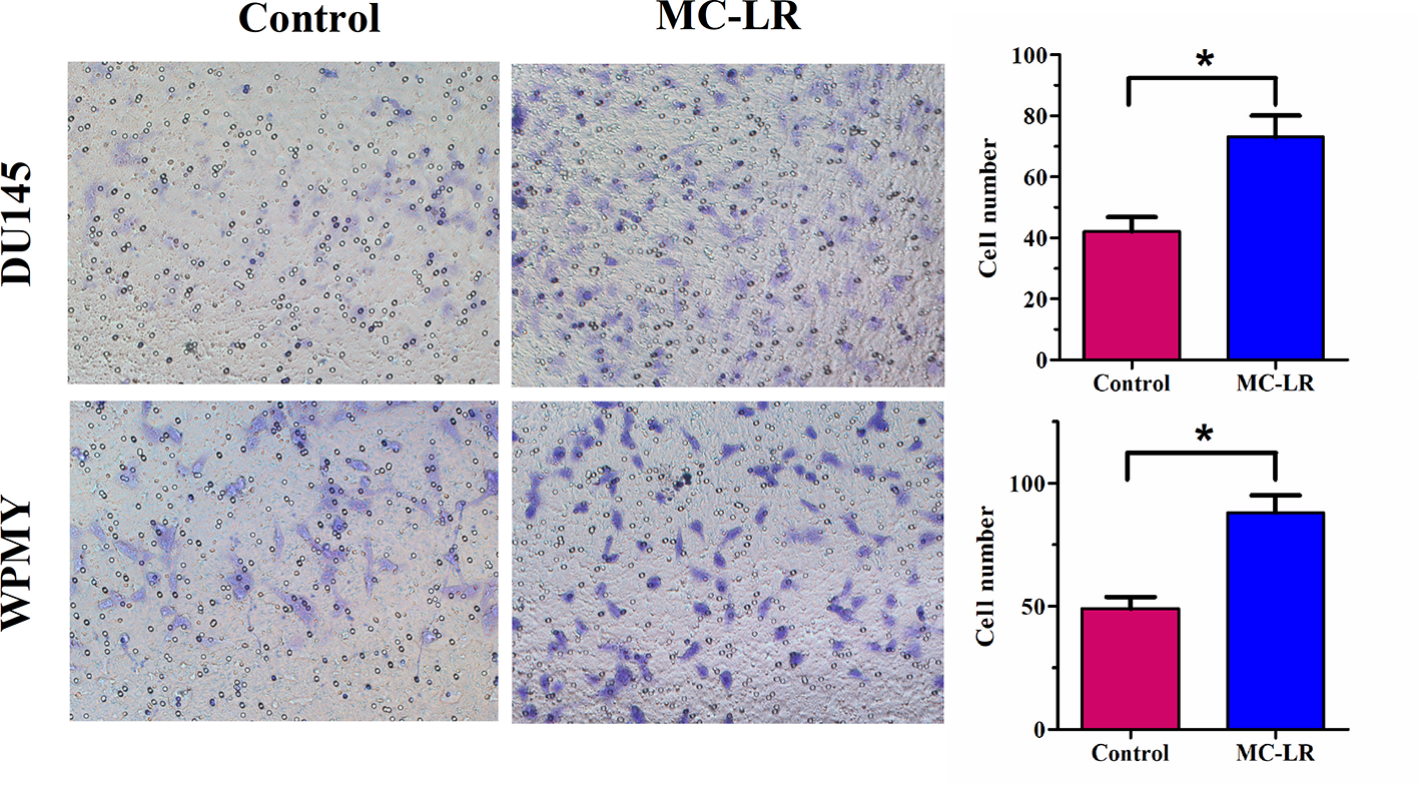
MC-LR promoted cell invasion in DU145 cells and WPMY cells. DU145 cells and WPMY cells were treated with 10 μM MC-LR for 24 h. The invasion ability was determined by transwell assay. The results are representative of three independent experiments. * *P* < 0.05. Error bars indicate SD.

It has been reported that MC-LR has potential carcinogenicity; therefore, transwell assay was performed to determine the effect of MC-LR on cell invasion. The results revealed that the invasion ability of the MC-LR treatment group was substantially reinforced compared with the untreated group in DU145 and WPMY cells (P < 0.05, **Figure 6**). Thus, these data demonstrated that MC-LR promoted cell invasion in both DU145 and WPMY cells.

## Discussion

In the present study, we investigated the influence of MC-LR on mechanical parameters, microfilament, and cell invasion in DU145 and WPMY cells. DU145 and WPMY cells were treated with 10 μM MC-LR, and then the cellular deformability and viscoelastic parameters were tested by the micropipette aspiration technique. The results showed that MC-LR increased the cellular deformability, reduced the cellular viscoelastic parameter values, and caused the cells to become softer. Moreover, the immunofluorescence of microfilament was performed, and then Western blot of microfilament-associated proteins was carried out. The results demonstrated that MC-LR induced microfilament reorganization and increased the phosphorylation level of microfilament-associated proteins. Finally, the effect of MC-LR on cell invasion was examined. The results revealed that MC-LR promoted cell invasion. Collectively, our results suggested that mechanical changes and microfilament reorganization were involved in MC-LR-promoted cell invasion in DU145 and WPMY cells.

Since the effect of MC-LR on DU145 and WPMY cells has not been reported, the appropriate concentration of MC-LR was screened. The results showed that MC-LR did not affect the relative cell viability of DU145 and WPMY cells at concentrations of 0.1–10 μM. Consistent with our results, Wang et al. treated Hep-2 cells with 0.5–10 μM MC-LR for 24 h. Their results suggested that the relative cell viability remained unchanged in the treated group compared with the untreated group (Wang et al., 2017). Also, MC-LR at 10 μM was used in several other studies (Zeng et al., 2015; Liu et al., 2016; Wang et al., 2017). Therefore, 10 μM MC-LR was selected for the subsequent experiments to obtain a more obvious effect in this study.

Micropipette aspiration is a useful technique for detecting the viscoelasticity of cells, such as colon cancer cells (Taranejoo et al., 2016), scleral fibroblasts (Wang and Chen, 2012), and chondrocytes (Zhang et al., 2008). Therefore, in the present study, the micropipette aspiration technique was used to investigate the effect of MC-LR on the mechanical properties of DU145 and WPMY cells. The results showed that MC-LR increased the deformability of the cells, reduced the cellular viscoelastic parameter values, and caused the cells to become softer. Additionally, DU145 cells showed higher deformability and weaker viscoelastic behavior than WPMY cells, respectively. Previous studies indicated that mechanical properties are the key parameters that are correlated with metastasis and invasion of cancer cells (Rother et al., 2014; Runge et al., 2014; Nguyen et al., 2016). Specifically, Wang et al. reported that the viscoelastic characteristics of breast cancer cells were substantially decreased, and the cancer cells became softer and more fluid than normal cells (Wang et al., 2016). Xie et al. analyzed different cancer cell lines, including human skin cancer cells (A2058), lung cancer cells (MSTO-211H), and hepatocellular carcinoma cells (Hep G2), and their results suggested that all cancer cells exhibited higher deformability and weaker viscoelastic behavior than the normal cells, respectively (Xie et al., 2019). Based on our results and others’ studies, we speculated that MC-LR could induce cells to alter their structures and functions and become softer, easier to deform, and potentially easier to metastasize. To our knowledge, this is the first time that the influence of MC-LR on cellular mechanical properties has been investigated, providing novel information to explain the carcinogenic mechanism of MC-LR.

Previous research reported that cellular mechanical behavior was associated with microfilament changes. As a result, cellular microfilament arrangement was further tested in this study. The results of immunofluorescence showed that MC-LR induced microfilament reorganization, leading to microfilament distribution on the cell surface and aggregation into bundles in DU145 and WPMY cells. Consistent with our study, Zeng et al. found that MC-LR caused aggregation of microfilament around the cell periphery in HL7702 cells (Zeng et al., 2015). In addition, Wang et al. observed that microfilaments were reconstructed, gathering at the cell edge and forming bundles, in MC-LR-treated Hep-2 cells (Wang et al., 2017). Other studies also demonstrated that MC-LR could cause microfilament reorganization in human liver cancer cells (SMMC-7721) (Wang et al., 2014) and neuroendocrine cells (PC12) (Meng et al., 2011). Additionally, it has been reported that cellular mechanical behavior is closely related to the components of the cytoskeleton, such as microfilament and microtubule (Chen et al., 2013; Pachenari et al., 2014). Further studies suggested that chemical drugs could cause cytoskeleton reorganization and then induce mechanical changes (Seyedpour et al., 2015; Li M. et al., 2016). Therefore, our results indicated that MC-LR could induce mechanical changes by microfilament reorganization in DU145 and WPMY cells.

Several studies have suggested that microfilament-associated proteins play key roles in maintaining the structure of microfilament (Wang et al., 2017). Therefore, microfilament-associated proteins need to be further investigated. Several relevant studies have been documented. For example, Zeng et al. reported that MC-LR induced hyperphosphorylation of VASP and ezrin in HL7702 cells but had no significant effect on their expression level (Zeng et al., 2015). The study of Wang et al. showed that the expression of p-VASP was up-regulated remarkably in SMMC-7721 cells treated with MC-LR (Wang et al., 2014). In addition, other studies found that ezrin and VASP were regulated by phosphorylation in MC-LR-treated cells, which could lead to microfilament rearrangement (Neisch and Fehon, 2011; Thomson et al., 2011; Zhou et al., 2015). Likewise, we detected the expression and phosphorylation of microfilament-associated proteins, and the results confirmed that the expression of p-VASP and p-ezrin was significantly upregulated in MC-LR treatment cells. However, the expression of VASP and ezrin remained unchanged. It is well known that VASP and ezrin are important microfilament-associated proteins, belonging to Ena/VASP family of adaptor proteins (Yu et al., 2015) and the ERM (Ezrin/Radixin/Moesin) protein family (Mcclatchey, 2014), respectively. Previous studies have demonstrated that the phosphorylation changes in VASP and ezrin induced microfilament rearrangement (Zhou et al., 2015; Wang et al., 2017). As a result, the MC-LR-induced increase in the phosphorylation level of microfilament-associated proteins that we found in our study could contribute to microfilament reorganization in DU145 and WPMY cells.

It has been reported that MC-LR can promote cell migration and metastasis (Pengfei et al., 2013; Wang et al., 2017). In more detail, Wang et al. showed that MC-LR accelerated Hep-2 cell migration (Wang et al., 2017). Xu et al. reported that MC-LR promoted MDA-MB-435 cell invasion and metastasis *via* the PI3-K/AKT signaling pathway (Pengfei et al., 2013). In this study, we found that MC-LR promoted cell invasion in DU145 and WPMY cells. Previous studies indicated that mechanical properties are correlated with the metastasis and invasion of cancer cells (Rother et al., 2014; Runge et al., 2014; Nguyen et al., 2016). In addition, many studies also suggested that microfilaments were functionally associated with cell invasion (Moon and Wynshaw-Boris, 2013; Du et al., 2015; Wang et al., 2017). In the present study, we demonstrated that MC-LR induced cellular mechanical changes and microfilament reorganization and promoted cell invasion. Based on our results and previous studies, we speculate that MC-LR promoted cell invasion by microfilament reorganization and mechanical changes in DU145 and WPMY cells. Relevant further studies will be performed to elucidate the molecular mechanism of MC-LR-promoted cell invasion.

## Conclusion

In conclusion, we demonstrated that mechanical changes and microfilament reorganization were involved in MC-LR-promoted cell invasion in DU145 and WPMY cells. Our results provided novel and useful information to explain the toxicological mechanism of MC-LR.

## Data Availability Statement

All datasets generated for this study are included in the article/**Supplementary Material**.

## Author Contributions

WF, ZP, and GuoW designed the experiments and wrote the paper. QZ, GuiW, and YX performed the experiments. ZG and ZL analyzed the data. All of the authors read and approved the final manuscript.

## Funding

The study was supported by the Natural Science Foundation of Shandong Province (ZR2014CL034, ZR2018MC015, ZR2019MA018), Medical and Health Development Plan of Shandong Province (2017WS058), Research and Development Plan of University in Shandong Province (J18KA120), National Natural Science Foundation of China (11802209), and Funds of the Shandong “Double Tops” Program.

## Conflict of Interest

The authors declare that the research was conducted in the absence of any commercial or financial relationships that could be construed as a potential conflict of interest.

## Supplementary Material

Data Sheet 1 MC-LR increased the phosphorylation level of microfilament-associated proteins in DU145 and WPMY cells. These photos (including ezrin, vasp and β-actin-1) were the original image files for the Western blot of **Figure 5A**. Other photos (including p-ezrin, p-vasp and β-actin-2) were the original image files for the Western blot of **Figure 5B**.
