# Supplementary figures and images for: Mechanical Changes and Microfilament Reorganization Involved in Microcystin-LR-Promoted Cell Invasion in DU145 and WPMY Cells
Source: Front Pharmacol. 2020 Feb 26;11:89. doi: 10.3389/fphar.2020.00089 (PMC7054891; doi:10.3389/fphar.2020.00089)

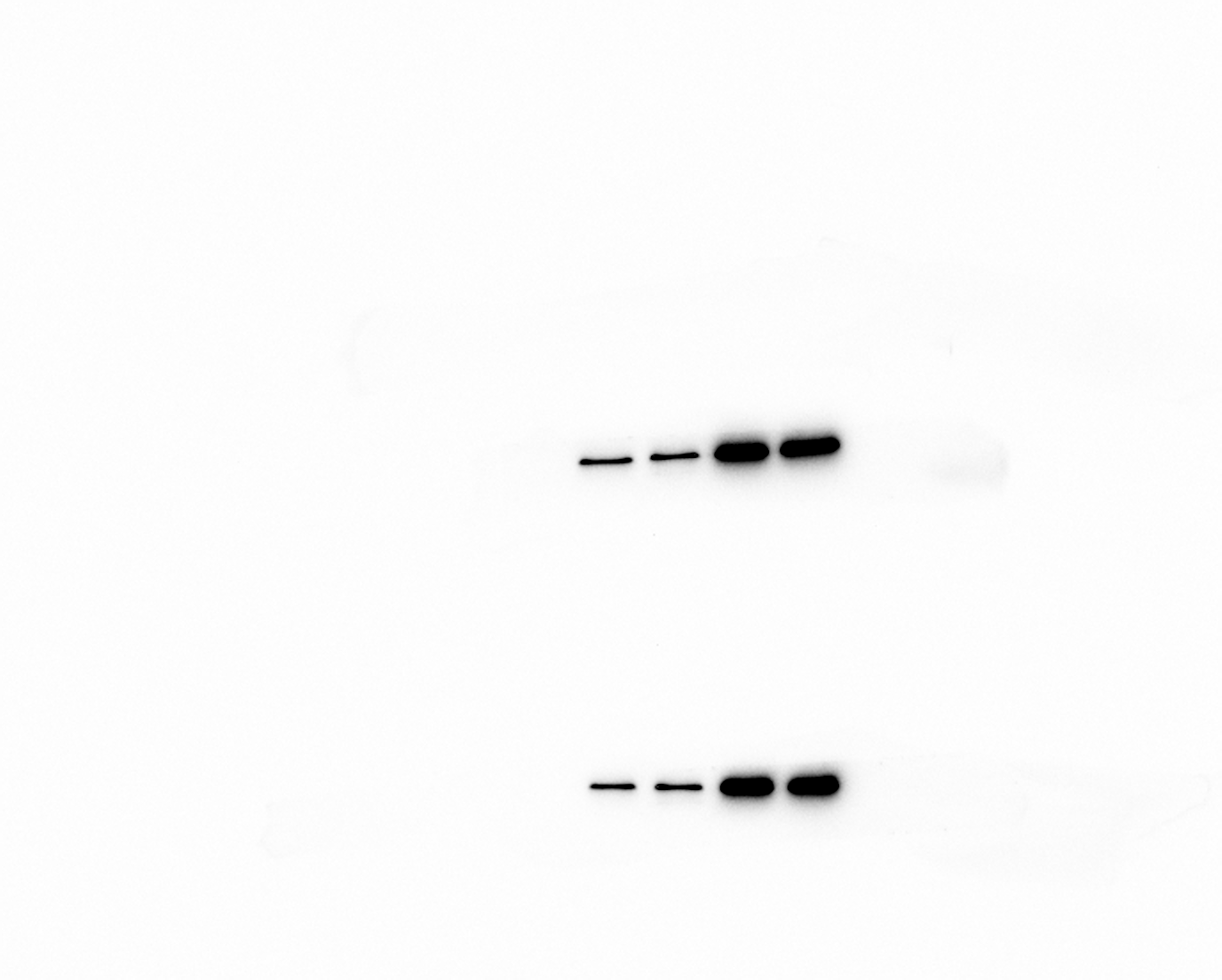

Supplement: Data Sheet 1 — MC-LR increased the phosphorylation level of microfilament-associated proteins in DU145 and WPMY cells. These photos (including ezrin, vasp and β-actin-1) were the original image files for the Western blot of Figure 5A . Other photos (including p-ezrin, p-vasp and β-actin-2) were the original image files for the Western blot of Figure 5B . [file DataSheet_1.zip › the original image files for the western blot/ezrin.Tif]

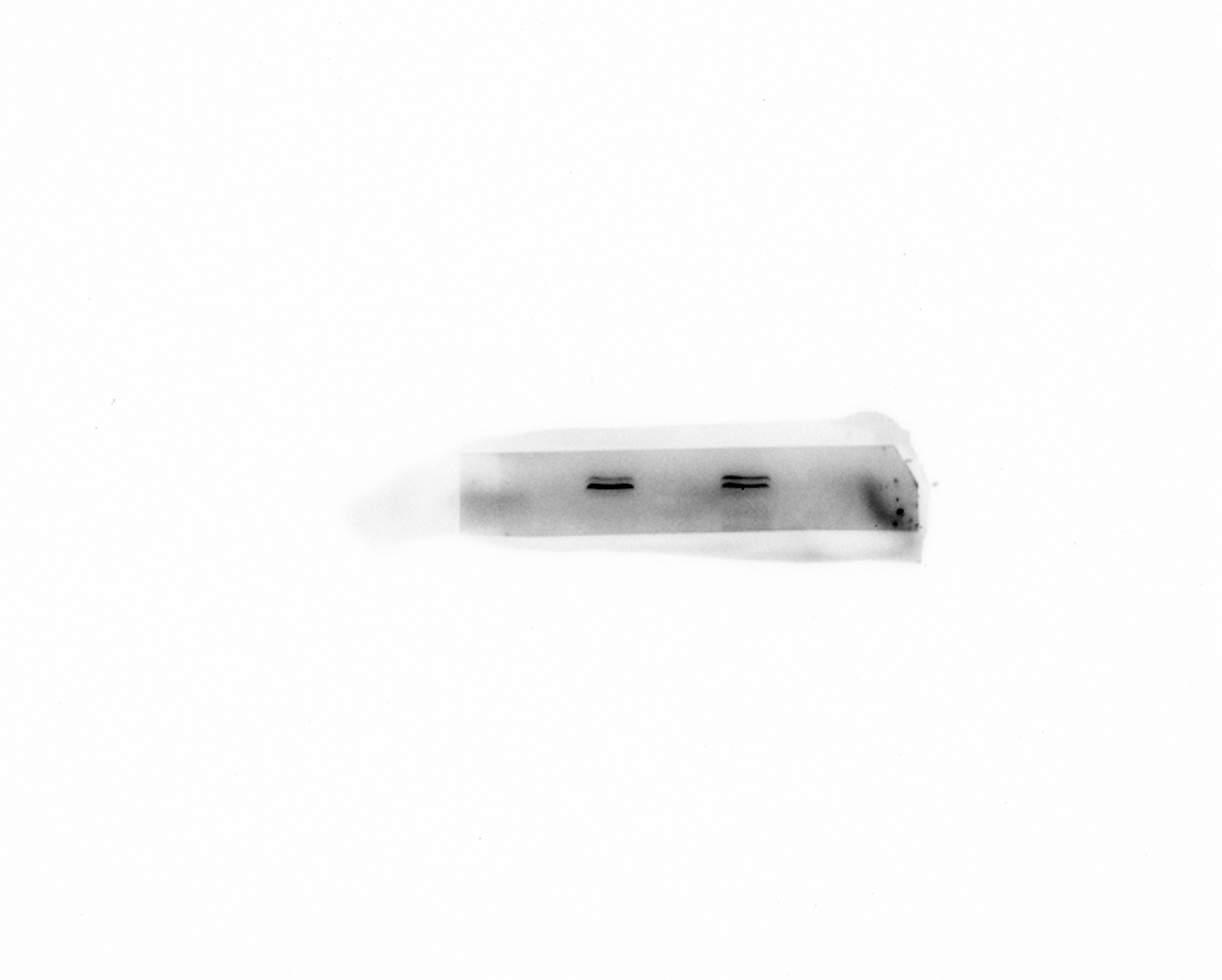

Supplement: Data Sheet 1 — MC-LR increased the phosphorylation level of microfilament-associated proteins in DU145 and WPMY cells. These photos (including ezrin, vasp and β-actin-1) were the original image files for the Western blot of Figure 5A . Other photos (including p-ezrin, p-vasp and β-actin-2) were the original image files for the Western blot of Figure 5B . [file DataSheet_1.zip › the original image files for the western blot/p-ezrin.Tif]

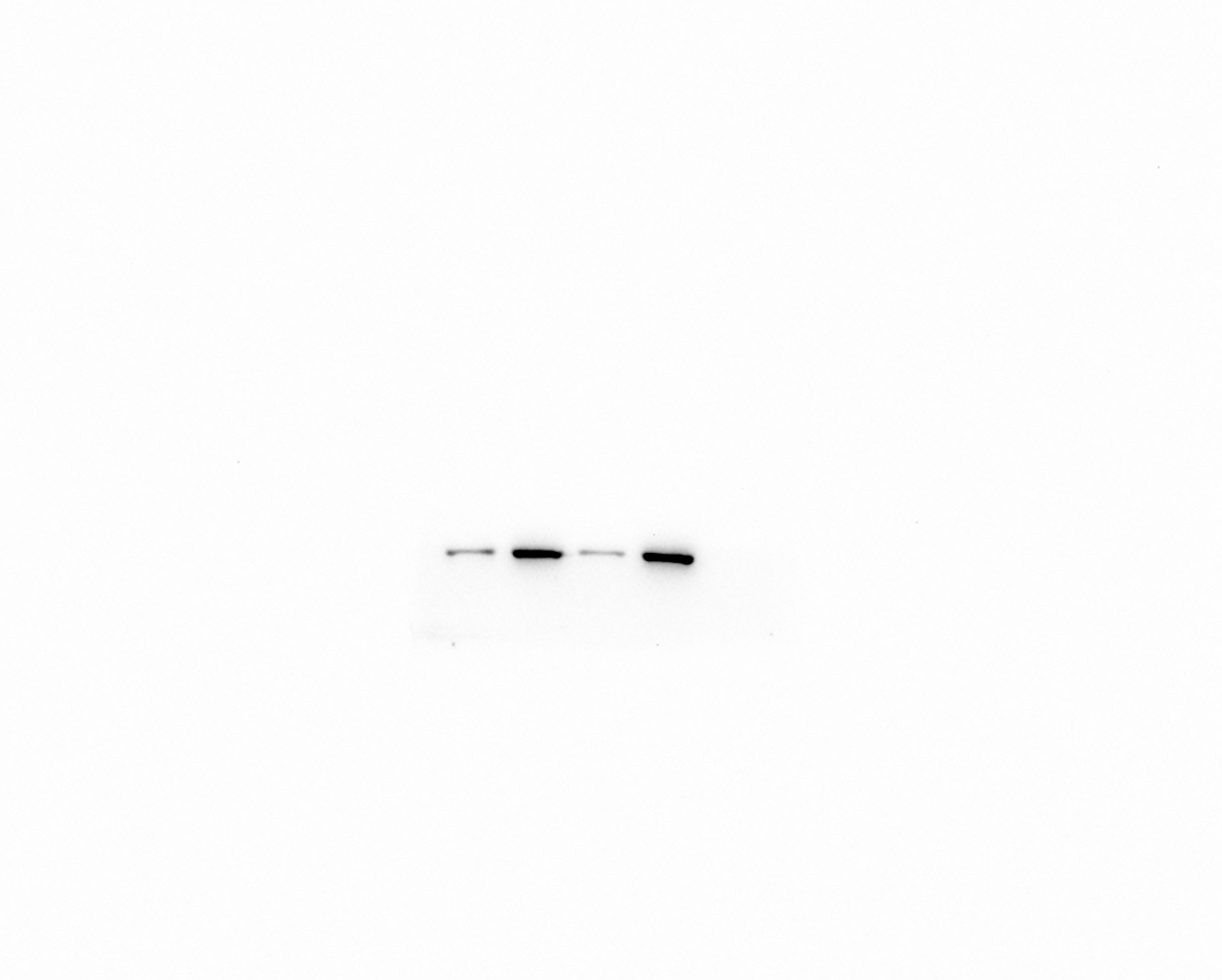

Supplement: Data Sheet 1 — MC-LR increased the phosphorylation level of microfilament-associated proteins in DU145 and WPMY cells. These photos (including ezrin, vasp and β-actin-1) were the original image files for the Western blot of Figure 5A . Other photos (including p-ezrin, p-vasp and β-actin-2) were the original image files for the Western blot of Figure 5B . [file DataSheet_1.zip › the original image files for the western blot/p-vasp.Tif]

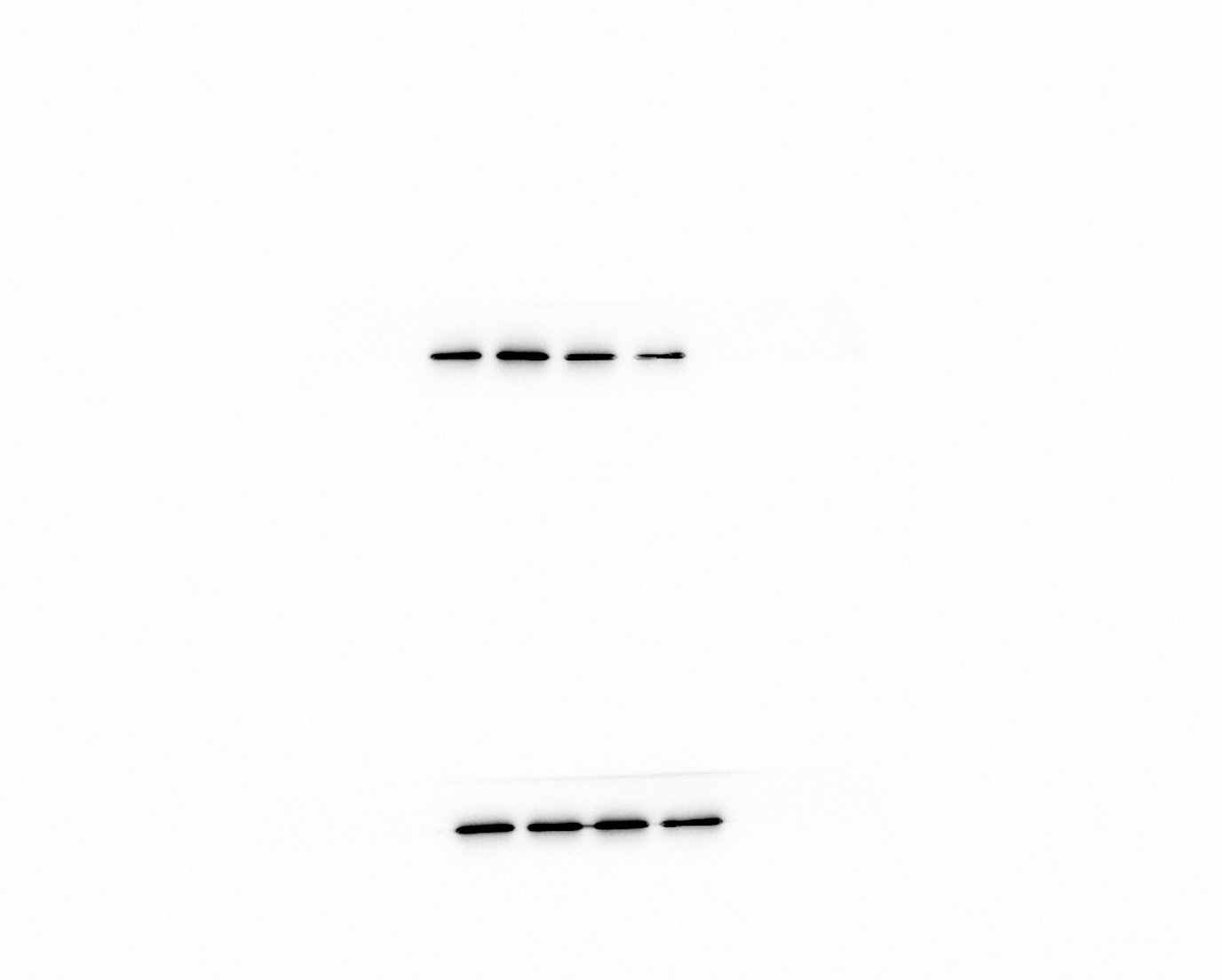

Supplement: Data Sheet 1 — MC-LR increased the phosphorylation level of microfilament-associated proteins in DU145 and WPMY cells. These photos (including ezrin, vasp and β-actin-1) were the original image files for the Western blot of Figure 5A . Other photos (including p-ezrin, p-vasp and β-actin-2) were the original image files for the Western blot of Figure 5B . [file DataSheet_1.zip › the original image files for the western blot/vasp.Tif]

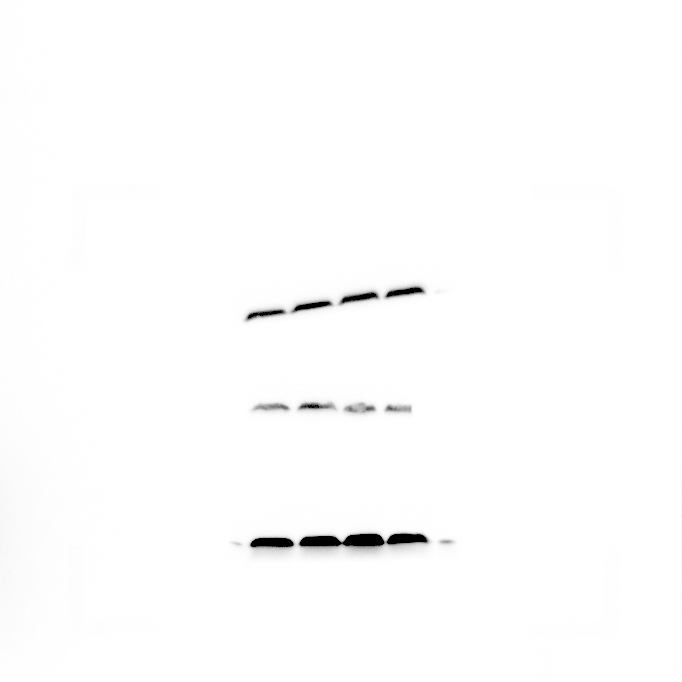

Supplement: Data Sheet 1 — MC-LR increased the phosphorylation level of microfilament-associated proteins in DU145 and WPMY cells. These photos (including ezrin, vasp and β-actin-1) were the original image files for the Western blot of Figure 5A . Other photos (including p-ezrin, p-vasp and β-actin-2) were the original image files for the Western blot of Figure 5B . [file DataSheet_1.zip › the original image files for the western blot/a┬-actin-1.tif]

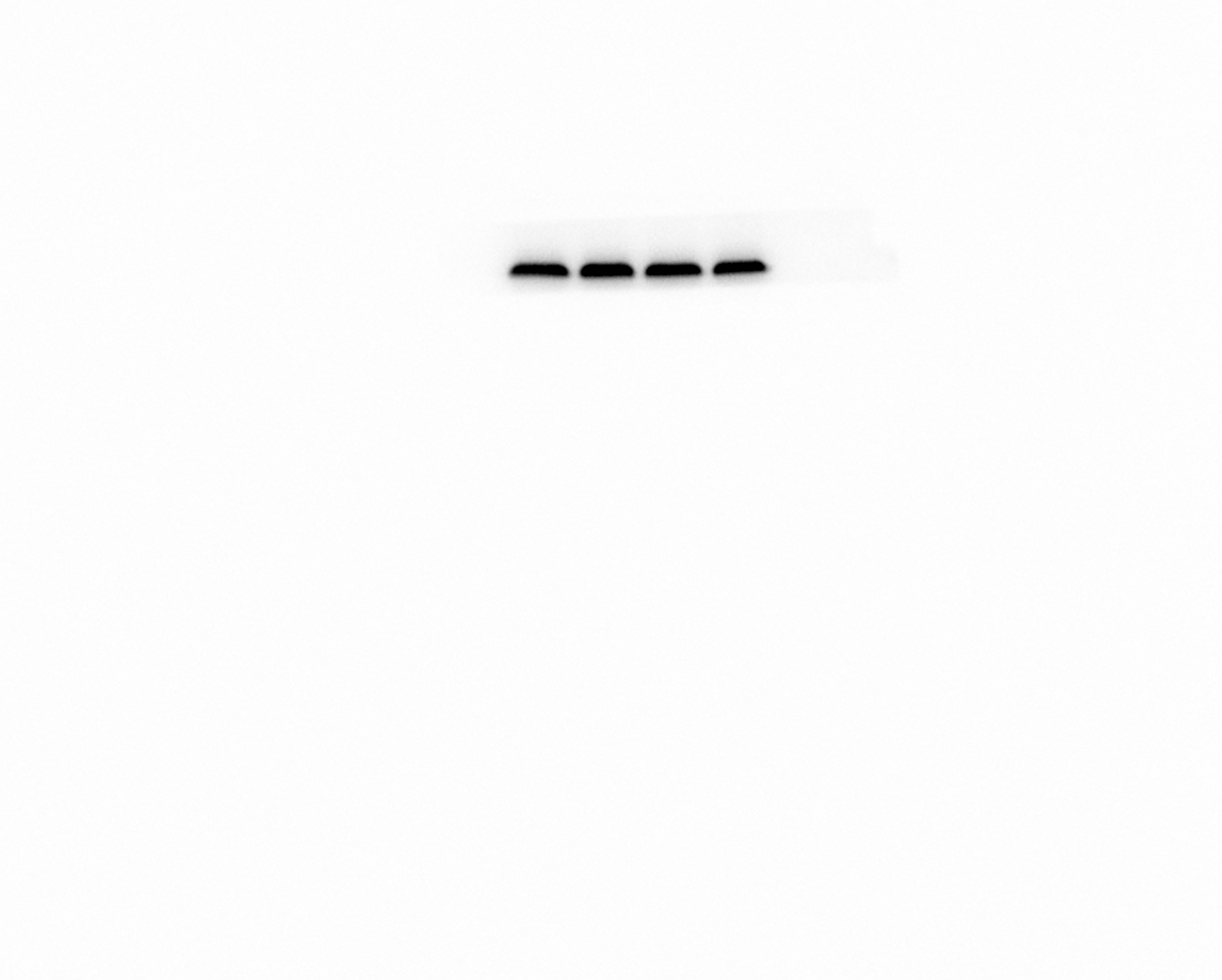

Supplement: Data Sheet 1 — MC-LR increased the phosphorylation level of microfilament-associated proteins in DU145 and WPMY cells. These photos (including ezrin, vasp and β-actin-1) were the original image files for the Western blot of Figure 5A . Other photos (including p-ezrin, p-vasp and β-actin-2) were the original image files for the Western blot of Figure 5B . [file DataSheet_1.zip › the original image files for the western blot/a┬-actin-2.Tif]
